# Author Correction: A library of 2D electronic material inks synthesized by liquid-metal-assisted intercalation of crystal powders

**DOI:** 10.1038/s41467-024-53248-8

**Published:** 2024-10-23

**Authors:** Shengqi Wang, Wenjie Li, Junying Xue, Jifeng Ge, Jing He, Junyang Hou, Yu Xie, Yuan Li, Hao Zhang, Zdeněk Sofer, Zhaoyang Lin

**Affiliations:** 1 Department of Chemistry, Engineering Research Center of Advanced Rare Earth Materials (Ministry of Education), Tsinghua University, 100084 Beijing, China; 2 Department of Inorganic Chemistry, University of Chemistry and Technology Prague, Technická 5, 166 28 Prague 6, Czech Republic

**Keywords:** Electronic devices, Electronic materials, Two-dimensional materials, Synthesis and processing

**Correction to:** *Nature Communications* 10.1038/s41467-024-50697-z, published online 29 July 2024

The original version of this Article omitted a reference to previous work in ‘*Sci. Adv*. **7**, eabe3767 (2021)’. This has been added as a new reference 38 to a new sentence on p. 2 of the Main Text: ‘A previous work also reported the incorporation of various nonmetallic materials such as graphite and graphene oxide into Gallium-based liquid metal and discussed the importance of surface oxide in the formation of putty-like composites^38^’. The numbers of the other references have been modified accordingly.

This has been corrected in the PDF and HTML versions of the Article.

