# Supplementary material for: A library of 2D electronic material inks synthesized by liquid-metal-assisted intercalation of crystal powders
Source: Nat Commun. 2024 Jul 29;15:6388. doi: 10.1038/s41467-024-50697-z (PMC11289403; doi:10.1038/s41467-024-50697-z)
Supplement: Supplementary file 1 — Supplementary Information [file 41467_2024_50697_MOESM1_ESM.pdf]

Supplementary Information for

A library of 2D electronic material inks synthesized by liquid-metal-assisted intercalation of crystal powders

Shengqi Wang[1], Wenjie Li[1], Junying Xue[1], Jifeng Ge[1], Jing He[1], Junyang Hou[1], Yu Xie[1], Yuan Li[1], Hao Zhang[1], Zdeněk Sofer[2] & Zhaoyang Lin[1*]

[1]Department of Chemistry, Engineering Research Center of Advanced Rare Earth Materials (Ministry of Education), Tsinghua University, Beijing, 100084, China.

[2]Department of Inorganic Chemistry, University of Chemistry and Technology Prague, Prague 6, 166 28 Czech Republic.

*To whom correspondence should be addressed.

Email: zlin@mail.tsinghua.edu.cn

Supplementary Figures

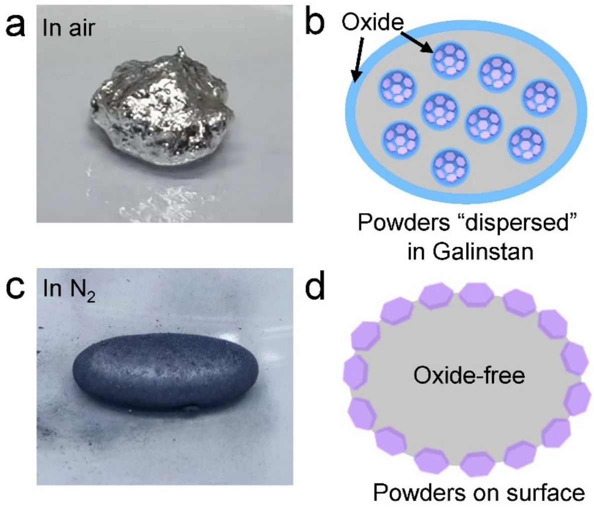

Supplementary Fig. 1 | The role of oxide in the dissolution of 2D crystal powders in Galinstan. a-d, Photographs and schematic illustration of the slurry mixture after continuously stirring the black powder and liquid metal for 10 min in air (a,b) and in N_2 environment (c,d), respectively. The slurry shows a metallic color in air, indicating that the pristine black powders are completely encased.

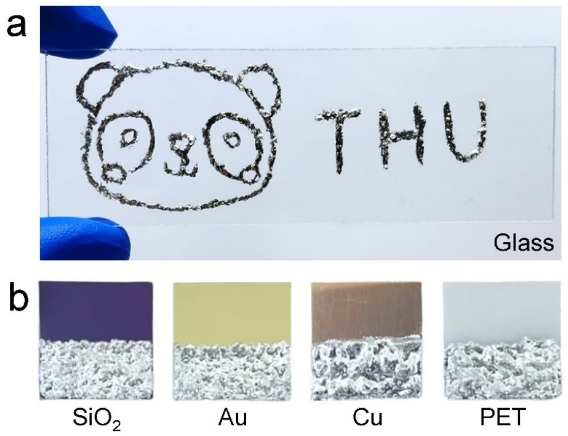

Supplementary Fig. 2 | Photographs of the mixture slurry of MoS_2 crystal powders and Galinstan coated on various substrates. a,b, The slurry that is coated and patterned on a variety of rigid and flexible substrates, including glass (a), silicon, gold film, copper plate, and PET (b).

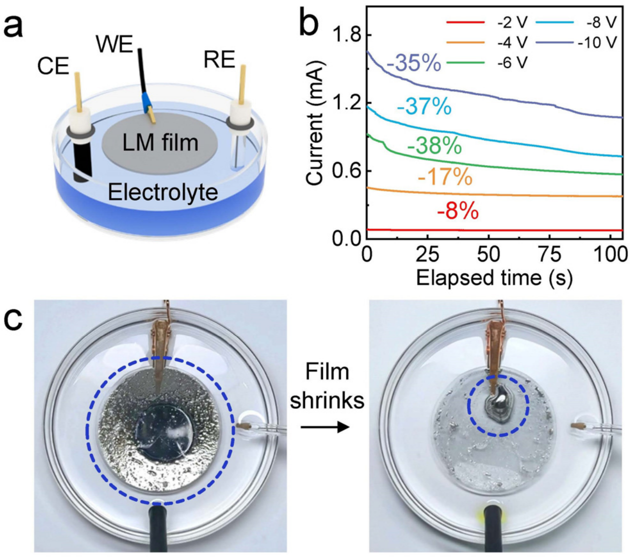

Supplementary Fig. 3 | Shrinking of liquid metal in THA^+/DMF solution under electrochemical potential. a, Schematic illustration of the electrolytic cell using Galinstan coated 2-inch glass as the working electrode. **b,** Chronoamperometry curves recorded at an applied voltage ranging from -2 to -10 V. The current drops significantly at high voltages (beyond -6 V) due to the shrinking of liquid metal and thus the reduced surface area of Galinstan. **c,** The photographs of Galinstan film shrinking into a droplet with the applied negative bias.

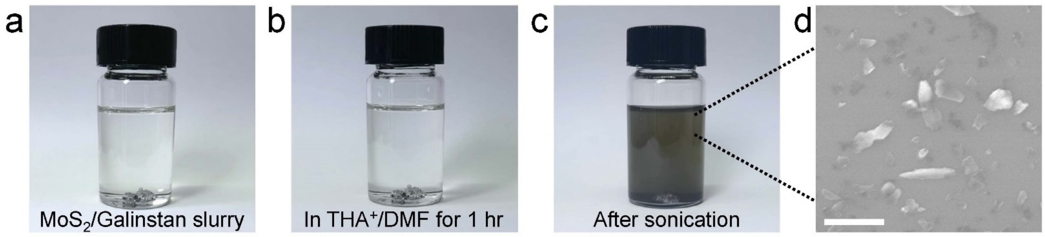

Supplementary Fig. 4 | Intercalation and exfoliation of MoS_2 crystals with THAB in the absence of external potential. a,b, Photographs of MoS_2/Galinstan slurry immersed in THA^+/DMF electrolyte for 0 hour (a) and 1 hour (b). No sign of intercalation or leaching of powders into solution was observed. **c,d,** Photograph of the obtained colloidal solution (c) and SEM image (d) of the products by directly sonicating the non-intercalated MoS_2/Galinstan slurry in DMF solution. Most of the crystals were not

exfoliated and stayed at the bottom of the vial. Scale bar, 1 μm.

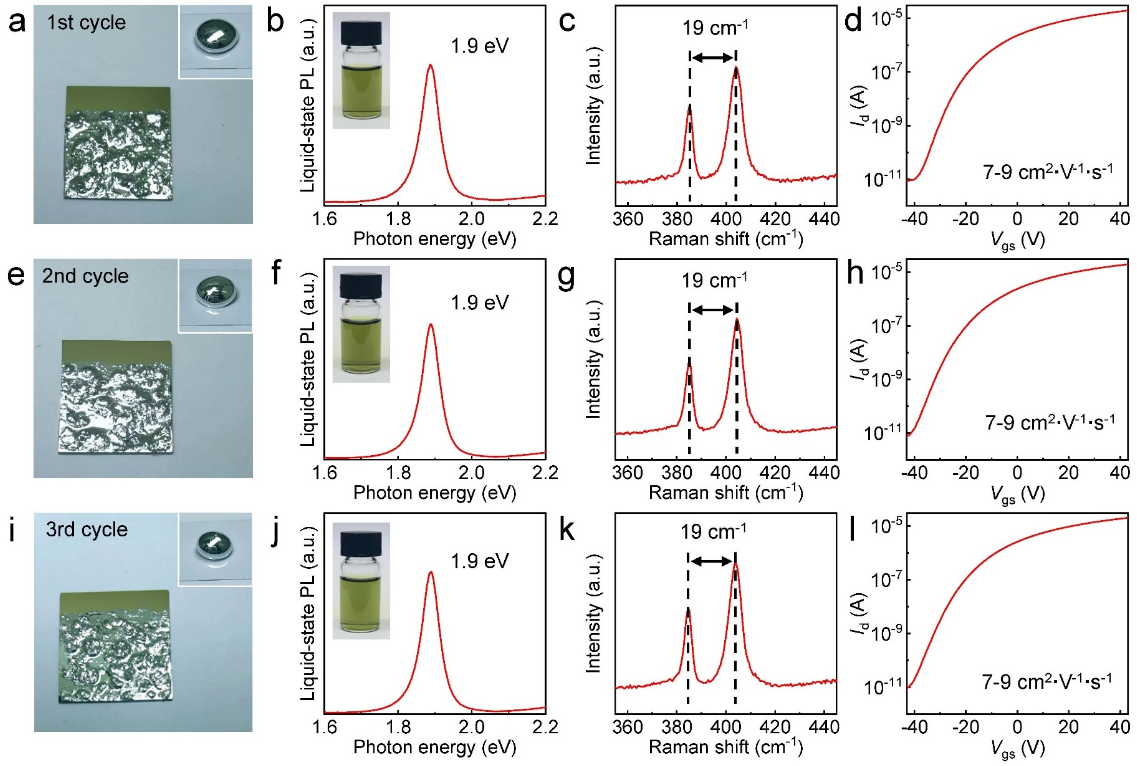

Supplementary Fig. 5 | The recyclability of liquid metal for the electrochemical intercalation. a-d, Photographs of Galinstan and slurry (a), liquid-state photoluminescence spectrum of the obtained ink solution (b), Raman spectrum of MoS_2 monolayer (c), and I_d–V_{gs} transfer characteristics of the prepared film (d), using fresh liquid metal for the 1st cycle of intercalation and exfoliation of MoS_2 powders. The term "a.u." denotes "arbitrary units". **e-h,** Photographs of Galinstan and slurry (e), liquid-state photoluminescence spectrum of the obtained ink solution (f), Raman spectrum of MoS_2 monolayer (g), and I_d–V_{gs} transfer characteristics of the prepared film (h), using recycled liquid metal for the 2nd cycle of intercalation and exfoliation of MoS_2 powders. **i-l,** Photographs of Galinstan and slurry (i), liquid-state photoluminescence spectrum of the obtained ink solution (j), Raman spectrum of MoS_2 monolayer (k), and I_d–V_{gs} transfer characteristics of the prepared film (l), using re-recycled liquid metal for the 3rd cycle of intercalation and exfoliation of MoS_2 powders. The applied V_{ds} is 1 V for all transfer curves.

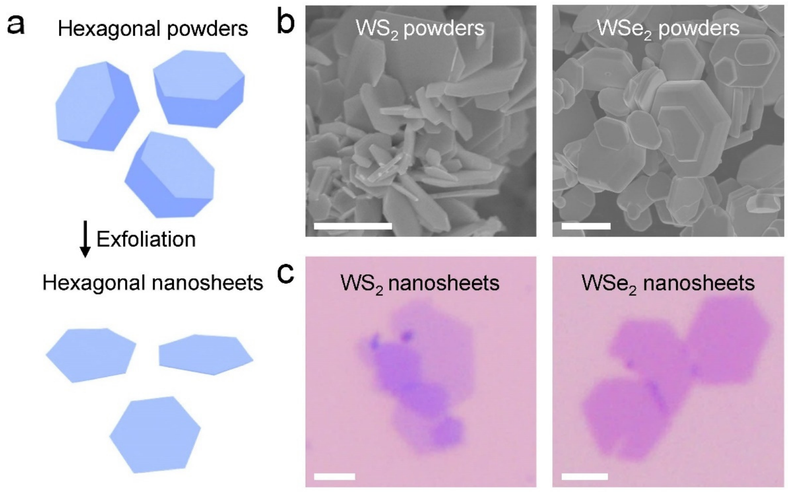

Supplementary Fig. 6 | Exfoliation of 2D nanosheets in a hexagonal shape. a, Schematic illustration of the electrochemical intercalation and exfoliation of hexagonal crystal powders to 2D

nanosheets with a regular hexagonal shape. **b,** SEM images of hexagonal WS_2 and WSe_2 powders used for the source materials for the electrochemical intercalation process. Scale bars, 2 μm (WS_2) and 5 μm (WSe_2). **c,** Optical images of the exfoliated WS_2 and WSe_2 hexagonal nanosheets. Scale bars, 2 μm.

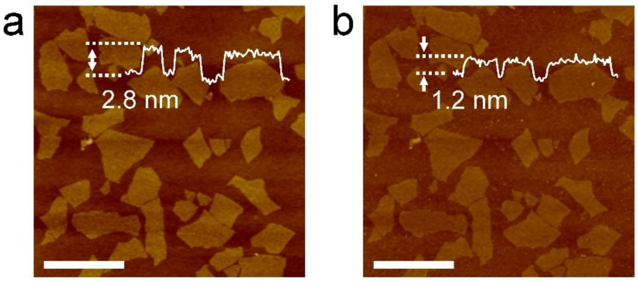

Supplementary Fig. 7 | Thickness characterization of MoS_2 monolayers before and after annealing. a,b, AFM images of MoS_2 monolayer nanosheets before (a) and after (b) annealing to show the removal of ligands. The apparent AFM thickness of the MoS_2 monolayer nanosheets is larger than the theoretical value of ~0.6 nm, despite other characterizations have confirmed the removal of organic molecules and restoration of pristine MoS_2 lattice. Scale bars, 2 μm.

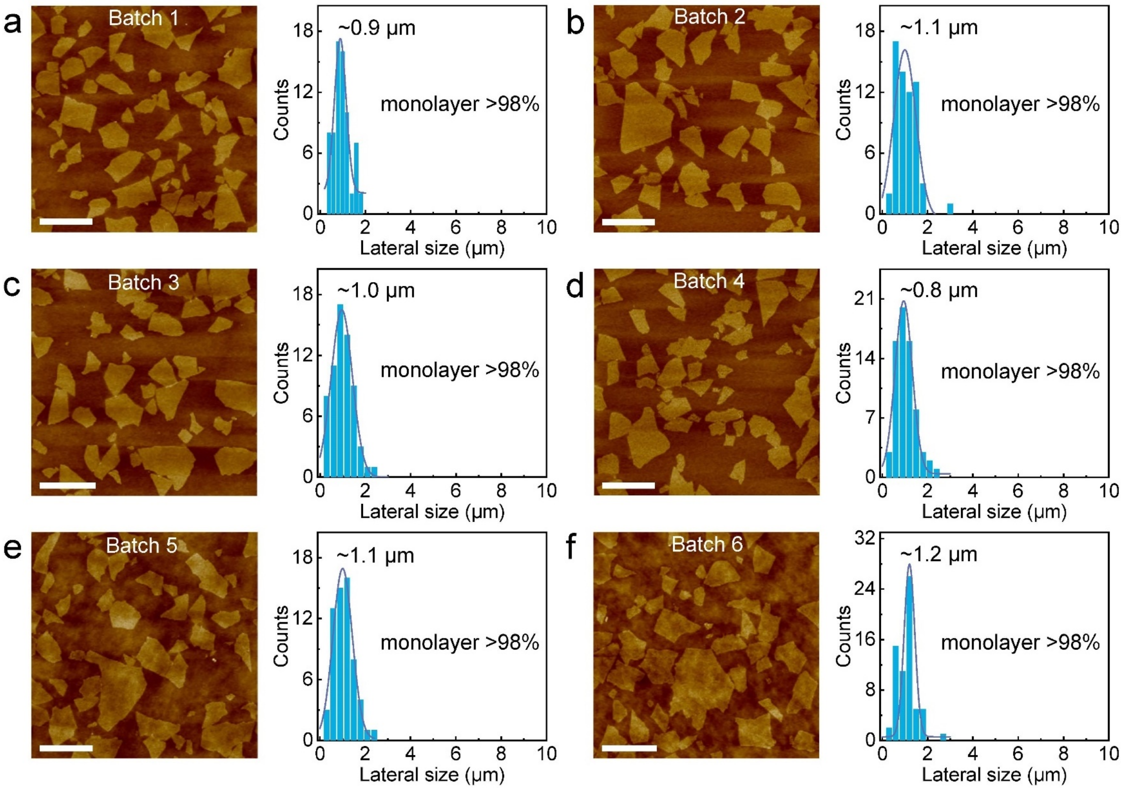

Supplementary Fig. 8 | AFM analyses of the 2D MoS_2 nanosheets obtained from six independent intercalation and exfoliation processes. a-f, AFM image and distribution of lateral size and thickness of exfoliated MoS_2 nanosheets prepared in six independent batches. The consistent nanosheet morphology suggests good batch-to-batch reproducibility. Scale bars, 2 μm.

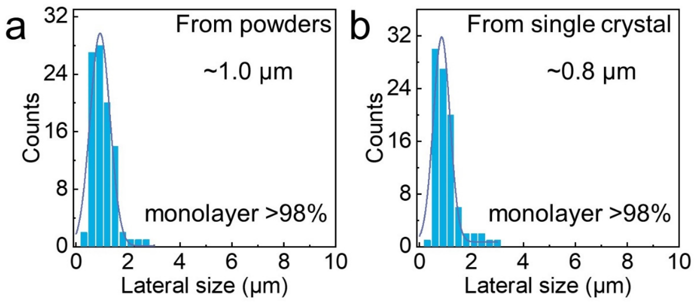

Supplementary Fig. 9 | Statistical analysis of the lateral size and thickness of the exfoliated MoS_2 nanosheets from crystal powders and bulk single crystal. a,b, Lateral size and thickness distribution of MoS_2 nanosheets exfoliated from crystal powders (a) and bulk single crystals (b).

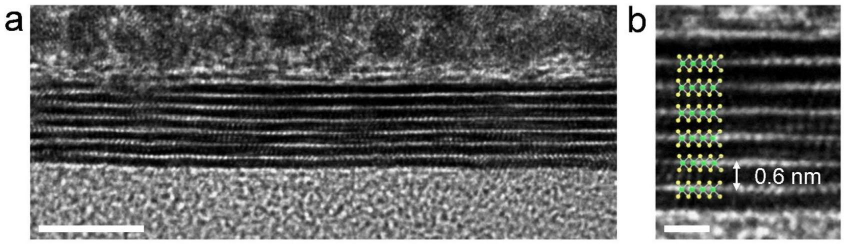

Supplementary Fig. 10 | Cross-sectional TEM analyses of annealed MoS_2 films assembled from exfoliated monolayers. The interlayer distance is ~0.6 nm which matches that in the pristine bulk crystal and thus suggests the complete removal of organic molecules between MoS_2 monolayers. Scale bars, 5 nm (a) and 1 nm (b).

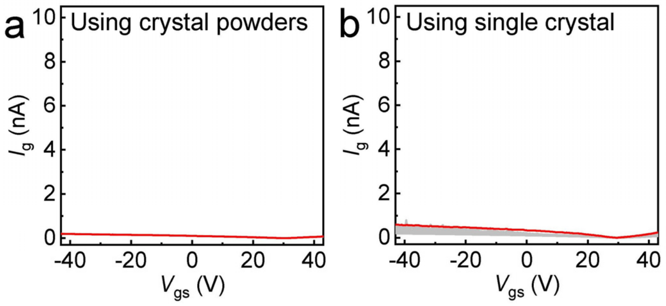

Supplementary Fig. 11 | Leakage current profile for MoS_2 transistors from exfoliation of crystal powders and bulk single crystal. a-b, I_g-V_{gs} leakage current of 20 individual MoS_2 transistors based on 2D nanosheets exfoliated from crystal powders (a) and large-piece single crystal (b). They correspond to the transfer curves in Fig. 2d and 2h in the main text.

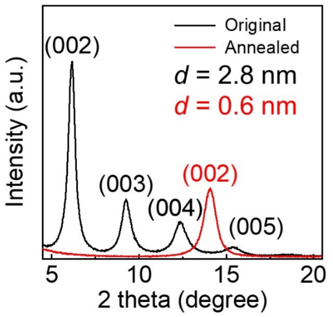

Supplementary Fig. 12 | XRD pattern of exfoliated MoS_2 nanosheets thin film before and after thermal annealing. The film by stacking exfoliated MoS_2 monolayers exhibits PVP/THAB superlattice structure with a lattice spacing of 2.8 nm. The disappearance of intrinsic MoS_2 (001) peak (d=0.6 nm)

suggests the absence of multilayer nanosheets and thus high monolayer purity. After annealing to remove organic molecules, this superlattice is converted to pure MoS_2 thin film with a lattice spacing of 0.6 nm. The term “a.u.” denotes “arbitrary units”.

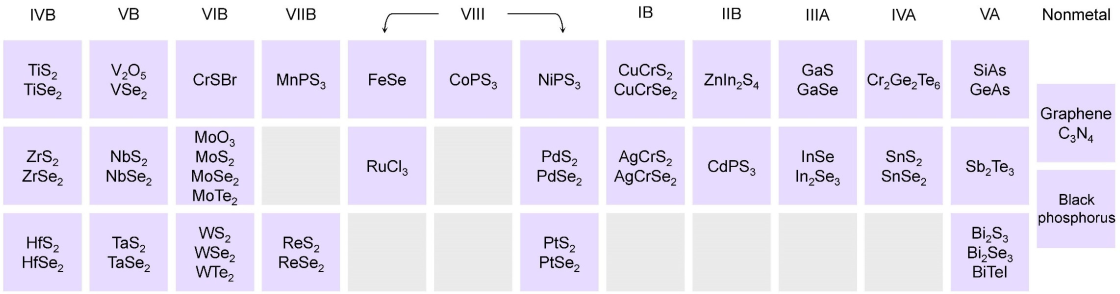

Supplementary Fig. 13 | The summary of representative layered crystal powders that can be processed with the powder-based intercalation assisted with liquid metal.

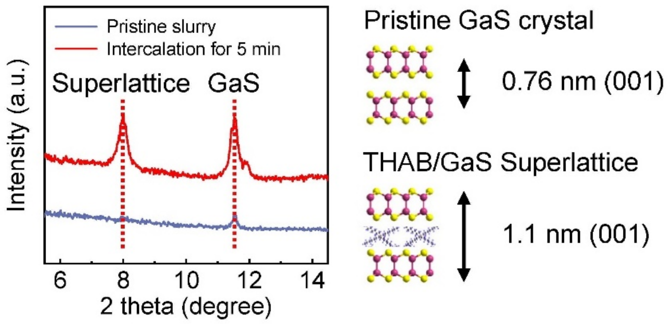

Supplementary Fig. 14 | XRD patterns of GaS mixture slurry before and after electrochemical molecular intercalation. Both the GaS crystal (~7.6 Å) and THAB/GaS hybrid superlattice (~11 Å) peaks grow stronger with the progression of the intercalation reaction.

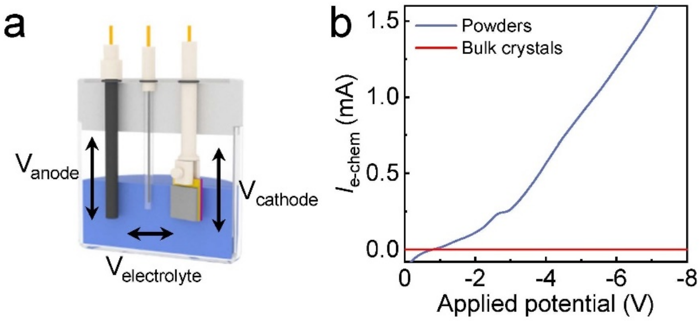

Supplementary Fig. 15 | Electrochemical analyses of GaS microcrystal slurry and bulk crystal. a, Schematic illustration of the potential drop at different parts of the electrochemical cell. **b,** Comparison of electrochemical current profiles when using a mixture slurry of microcrystal and bulk crystal.

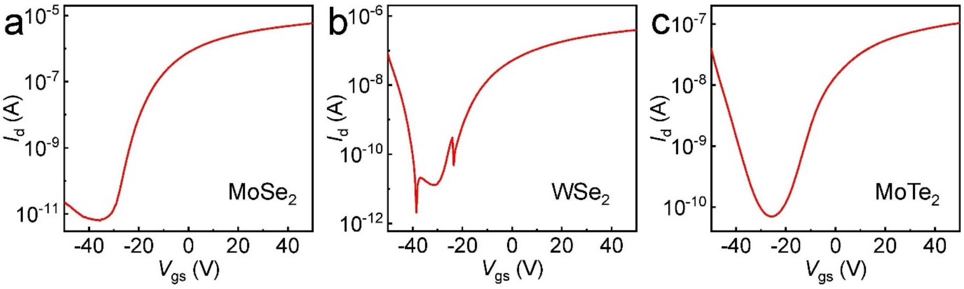

Supplementary Fig. 16 | I_d-V_{gs} transfer characteristics of transistors based on typical bipolar TMDs. a, $MoSe_2$. **b,** WSe_2, **c,** $MoTe_2$. These nanosheet thin films were annealed at 200 °C for 1 h in an

argon atmosphere. All devices were fabricated on 100 nm SiO_2/Si substrate and measured with V_{ds} of 1 V.

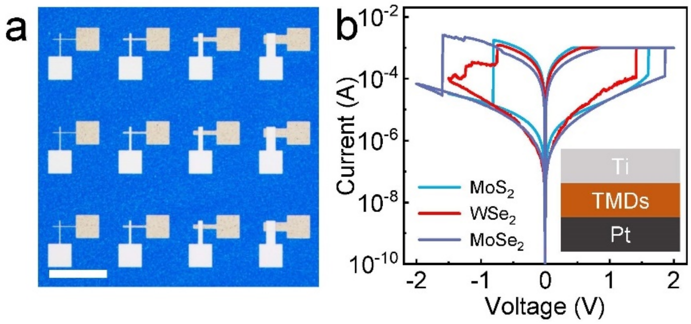

Supplementary Fig. 17 | Solution-processable memristors based on the exfoliated TMDs. a, Optical image of the MoS_2 memristors. Scale bars, 200 μm. **b,** The representative *I*-*V* curves of MoS_2, $MoSe_2$, and WSe_2 memristors in a vertical structure using asymmetric bottom Pt and top Ti contact electrodes.

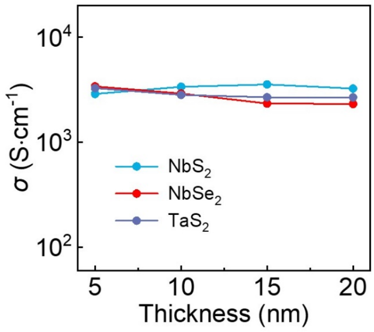

Supplementary Fig. 18 | Electrical characterizations of 2D metallic TMD thin films based on exfoliated nanosheets. The electrical conductivity of spin-coated NbS_2, $NbSe_2$, and TaS_2 thin films was measured at room temperature with a film thickness of 5-20 nm.

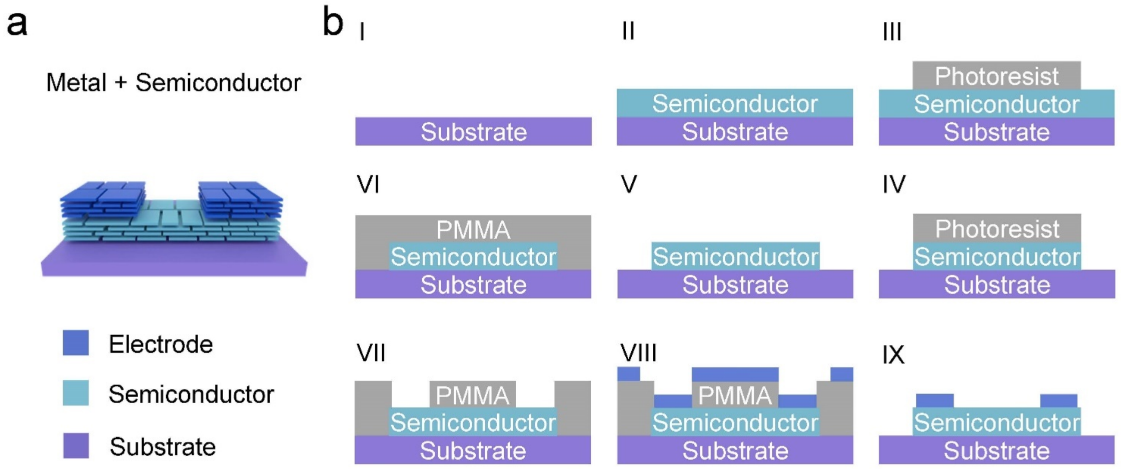

Supplementary Fig. 19 | Solution-processable integration of 2D metals and 2D semiconductors. a,b, Schematic illustration of the structure (a) and fabrication process (b) of back-gate thin-film transistors consisting of 2D metal and 2D semiconductor thin films.

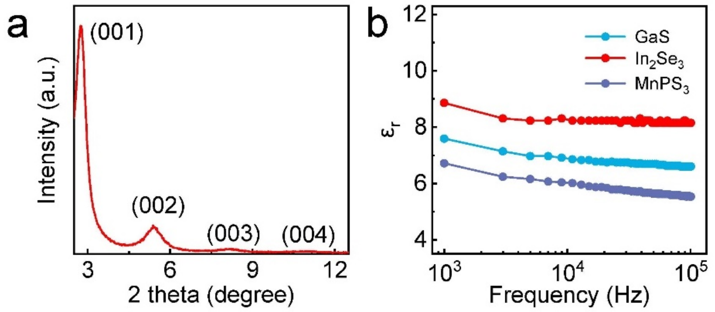

Supplementary Fig. 20 | Characterizations of the solution-processable 2D dielectrics. a, XRD patterns of GaS films after annealing at 200 °C for 1 hour. The preserved superlattice structure suggests good thermal stability at this temperature, which differs from the PVP/MoS_2 superlattice. **b,** Frequency dependence of the dielectric constant of GaS, In_2Se_3, and $MnPS_3$ films.

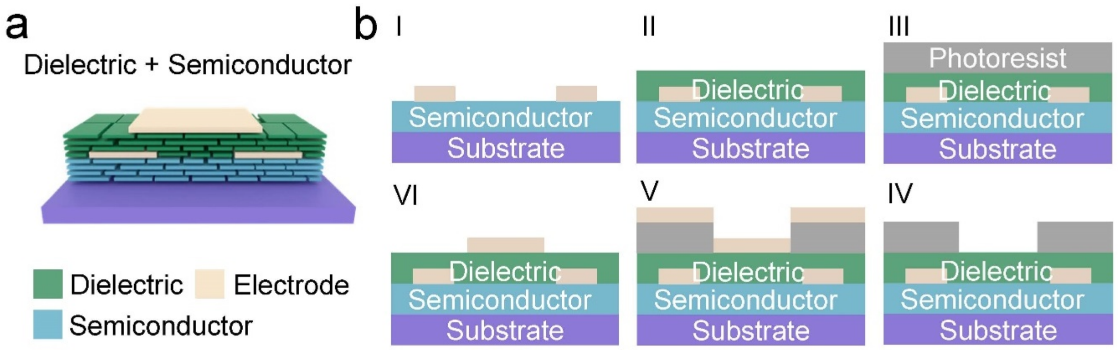

Supplementary Fig. 21 | Solution-processable integration of 2D dielectrics and 2D semiconductors. a,b, Schematic illustration of the structure (a) and fabrication process (b) of the top-gate thin-film transistor consisting of 2D dielectric and 2D semiconductor thin films with evaporated metal electrodes.

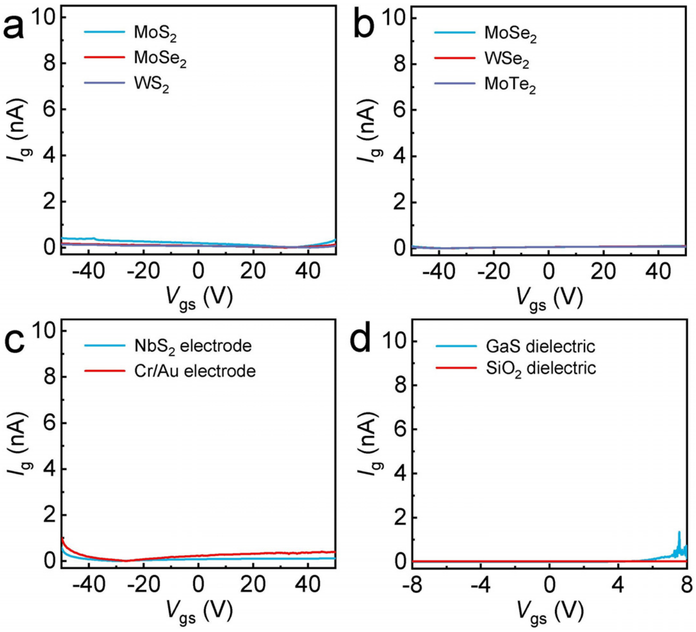

Supplementary Fig. 22 | Leakage current profiles for various solution-processable 2D transistors. a,b, I_g-V_{gs} leakage current of transistors based on n-type semiconducting MoS_2, $MoSe_2$, and WS_2 thin films (a) and p-type semiconducting $MoSe_2$, WSe_2, and $MoTe_2$ thin films (b). They correspond to the transfer curves in Fig. 5b and 5c of the main text. **c,** I_g-V_{gs} leakage current of MoS_2

transistors with solution-processable NbS_2 and evaporated Cr/Au electrodes. It corresponds to the transfer curves in Fig. 5f of the main text. **d,** I_g-V_{gs} leakage current of MoS_2 transistors with 40-nm-thick solution-processable 2D GaS and conventional 100-nm-thick thermal SiO_2 gate dielectric. It corresponds to the transfer curves in Fig. 5i of the main text.
